# Performance Optimization of Force Feedback Control System in Virtual Vascular Intervention Surgery

**DOI:** 10.1155/2014/673415

**Published:** 2014-09-01

**Authors:** Zhi Hu, Ping Cai, Peng Qin, Le Xie

**Affiliations:** ^1^School of Electronic, Information and Electrical Engineering, Shanghai Jiao Tong University, Shanghai 200240, China; ^2^Digital Manufacturing Technology Center, Shanghai Jiao Tong University, Shanghai 200240, China

## Abstract

In virtual surgery of minimally invasive vascular intervention, the force feedback is transmitted through the flexible guide wire. The disturbance caused by the flexible deformation would affect the fidelity of the VR (virtual reality) training. SMC (sliding mode control) strategy with delayed-output observer is adopted to suppress the effect of flexible deformation. In this study, the control performance of the strategy is assessed when the length of guide wire between actuator and the operating point changes. The performance assessment results demonstrate the effectiveness of the proposed method and find the optimal length of guide wire for the force feedback control.

## 1. Introduction

Virtual surgery is an effective training method to help novice surgeons to avoid operative errors during a real surgical process [1–3]. The application of the virtual reality training approach is not efficient because few methods can deliver haptic feedback that help trainees to feel fully fidelity [4].

Image information has emerged quickly enough, thanks to the development of *GPU* technology [5], while the real-time performance of force feedback system in virtual surgery is far from satisfactory because of the existence of system lag. When the force transmitting device is flexible, the situation of system lag would be even worse [6, 7].

Interventional cardiovascular surgery is a complex surgery. Before the surgery, doctors need to conduct a large number of surgical trainings. In recent decades, the design of VR simulator of vascular intervention surgery has aroused interests of engineering. The Simbionics company in America developed the *ANGIO* Mentor, and it generates the friction between the eccentric wheel and surgical instruments to provide the feedback force [8]. The Mentice company in Switzerland developed the VR simulator of Mentice *VIST*, and it enables the activation of the force feedback through application of pressure to surgical instruments [9]. The VR simulator of Simantha is developed by the Medical Simulation Corporation in the *USA*; the force feedback device of this simulator is located in a patient model [10]. Some scientists used the spring piece to create force feedback in the VR training system [11].

In above simulators of minimally invasive vascular intervention, there is a problem that the flexible deformation of the guide wire would lead to disturbance in force feedback transmission [12–15]. The response lag and force error caused by flexible deformation would make it hard to ensure fidelity by rendering the force feedback in actuator [13, 14].

To solve this problem, researchers generally use the passage of guide wire to simulate real vessel [10], while it cannot ensure the fidelity of force feedback [7, 16]. In this study, we adopt a control strategy to enhance the fidelity and assess the control performance when the length of guide wire between actuator and the operating point changes.

Several control strategies can be used in the force feedback system. However, some limitations exist. For instance, the output feedback variable structure control can suppress plant model uncertainty efficiently; however, the control input is too large during the transient stage, which may violate saturation constraint [17]. The loop-shaping method is used to improve performance and stability of force feedback, while nonlinearities can affect the behavior of the controller away from the nominal operating point [18]. Force/position control strategy is an effective way to help the operator to interact with the virtual environment, while it is also affected by the uncertainties of dynamical model and environment stiffness [19, 20].

In this study, the SMC (sliding mode control) strategy with delayed-output observer is used to suppress the effects of system lags on system fidelity while maintaining system robustness. As the length of guide wire between actuator and operating point is different, the disturbance of flexible deformation is related to the length of guide wire between the actuator and the operating point. The control performance needs to be assessed when the length of guide wire changes. And the optimal length of guide wire can be determined through the performance assessment.

The rest of the paper is organized as follows. In Section 2, we analyse the control system of the virtual minimally invasive vascular intervention and the flexible deformation of the guide wire. In Section 3, we design the strategy of SMC with delayed-output observer to suppress the effect of flexible deformation and analyse its performance in the force feedback control. In Section 4, we assess the performance of the force feedback control and search for the optimal length of guide wire between the actuator and human hand. And, in Section 5, we draw the conclusion.

## 2. Virtual Minimally Invasive Vascular Intervention and the Flexible Deformation

**Figure 1:**
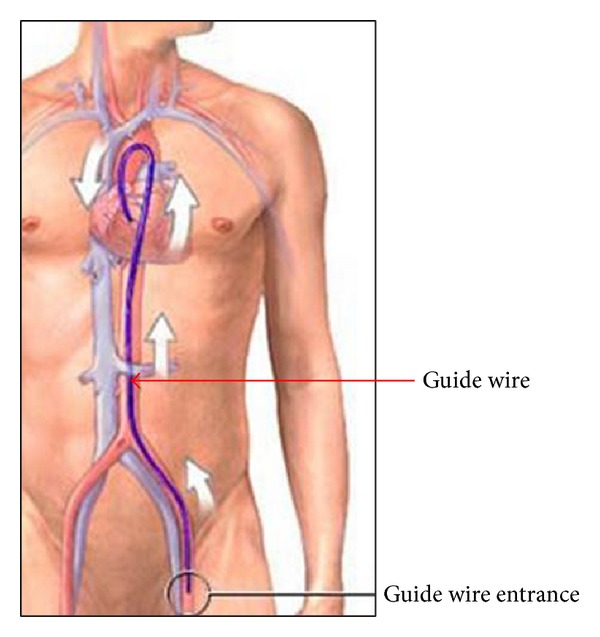
Minimally invasive vascular intervention.

Intracoronary Stenting surgery is performed to treat the stenosis or blockage of vessels [21], as shown in Figure 1. (Figure is adapted and redrawn from [22].)

There are several branches along the path of the vessels; the surgeon needs to skillfully operate the guide wire to reach the target place. One important requirement for the surgeon is to avoid producing too much resistance force between the guide wire and the walls of vessels; otherwise, the thin and delicate walls of vessels may be damaged by the guide wire.

### 2.1. Force Feedback Control System in Virtual Surgery of Minimally Invasive Vascular Intervention

**Figure 2:**
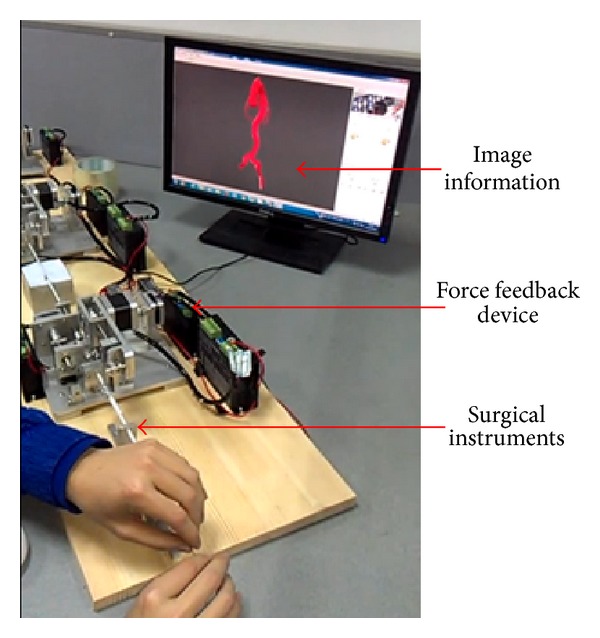
Virtual reality simulator of minimally invasive vascular intervention surgery.

In the force feedback control system of VR simulator, the input of the force feedback control represents resistance of the vessels in real surgery. The feedback force felt by the human hands is generated by actuators and it is transfered via surgical instruments (the guide wire, guide catheter, the thin guide wire, and ballon catheter). The VR simulator renders image information on the computer monitor, while the force feedback is provided by a device. Novice surgeons could be trained through the virtual surgery with the image and force feedback, as shown in Figure 2.

**Figure 3:**
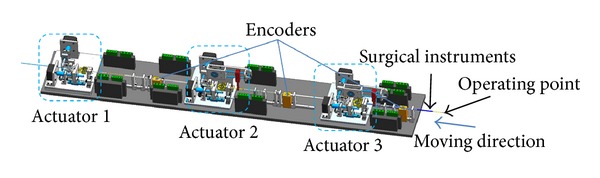
Force feedback device of minimally invasive vascular intervention; Actuator 1 creates the feedback force of the guide catheter and the thin guide wire; Actuator 2 creates the feedback force of the balloon catheter; Actuator 3 creates the feedback force of the guide wire.

The displacement of the surgical instruments is measured by encoders. The three actuators generate the force feedback of the surgical instruments separately. The VR training requires the force feedback to be the same as the real surgery.  Figure 3 shows the complete VR device.

**Figure 4:**
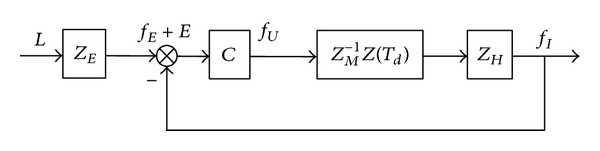
Force feedback control system of virtual surgery.

The guide wire has two degrees of freedom (DOF): forward-backward movement and rotation. The guide wire in the virtual surgery is flexible. In the forward-backward direction, the deformation of the guide wire between the Actuator 3 and the operating point of human hand needs to be taken into consideration [13]. The force feedback control system is shown in Figure 4.

*L* is the displacement of the force transmitting device, which is measured by the encoder. *Z*_*E*_ is the virtual environment of the vessel. *f*_*E*_ is the expected force feedback of the system. *C* is the control algorithm. *f*_*U*_ is the force given by the actuator. *f*_*I*_ is the measured interaction force. *E* is the error between *f*_*E*_ and *f*_*I*_. *Z*(*T*_*d*_) describes the effect of flexible deformation of the guide wire and *T*_*d*_ is the response delay, which will be discussed in Section 2.2. *Z*_*H*_ is the impedance of force sensor. Consider

(1) $$\begin{array}{c} Z_{H}=M_{H}s^{2}+B_{H}s+K_{H}, \end{array}$$

where *M*_*H*_, *B*_*H*_, and *K*_*H*_ are separately mass, damping, and stiffness parameters of the force sensor [23, 24]. *s* is the Laplace variable. *Z*_*M*_ is the mechanical impedance of the human-machine interaction interface. Consider

(2) $$\begin{array}{c} Z_{M}=M_{M}s^{2}+B_{M}s+K_{M}, \end{array}$$

where *M*_*M*_, *B*_*M*_, and *K*_*M*_ are separately mass, damping, and stiffness parameters of the mechanical impedance. As shown in Figure 4, the transfer function relating actuator force *f*_*U*_ with the measured interaction force *f*_*I*_ is as follows:

(3) p(s)=fIfU=ZM−1(s)ZH(s)Z(Td)=MHs2+BHs+KHMMs2+BMs+KMZ(Td).

There are parameter variations and response lags in the system. They would affect the force feedback control.

### 2.2. Flexible Deformation of the Guide Wire

**Figure 5:**
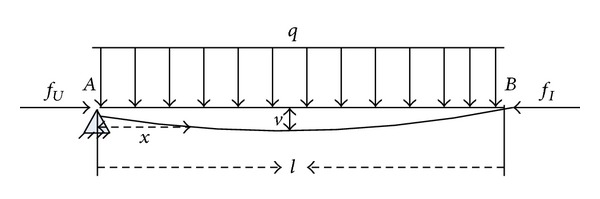
Force status of the guide wire.

In Intracoronary Stenting surgery [21], the guide wire may bend in the vessel. The flexible deformation would cause disturbance as there is energy stored [7]. The force status of the guide wire is shown in Figure 5.

The guide wire is under uniformly distributed load *q*, where *q* is the force per unit length due to gravity of the guide wire. *l* is the length of guide wire between actuator and the operating point. *v* is the deflection of the guide wire. *A* is the location of the Actuator 3 (as shown in Figure 3). *B* is the operating point of human hand (as shown in Figure 3). The stored energy caused by the bending moment is as follows [25]:

(4) $$\begin{array}{c} W=\frac{1}{2}\overset{l}{\underset{0}{\int }}\frac{M^{2}\left(x\right)}{EI}dx, \end{array}$$

where *x* is the displacement from *A* in the guide wire. *E* and *I* are separately elastic modulus and polar moment of inertia of the guide wire. *M* is the bending moment. Based on the dynamical equation, it can be shown that

(5) $$\begin{array}{c} EI\frac{d}{dx}\left(\frac{dv\left(x\right)}{dx}\right)=M\left(x\right)=f_{U}v\left(x\right)+\frac{1}{2}qx^{2}, \end{array}$$

where *v* is the deflection of the guide wire. When (0 < *x* < *l*/2), the solution of the equation is as follows:

(6) $$\begin{array}{c} v\left(x\right)=\frac{qx^{2}}{2f_{U}}\left(\cos\sqrt{\frac{f_{U}}{EI}}x-1\right). \end{array}$$

Using the Lagrange equation, it can be shown that

(7) $$\begin{array}{c} \frac{d}{dt}\left(\frac{\partial \left(W+W^{1}\right)}{\partial \dot{v}}\right)-\frac{\partial \left(W+W^{1}\right)}{\partial v}=Q, \end{array}$$

where *W* ^1^ is the kinetic energy of the guide wire and *Q* is the generalized force. Consider

(8) $$\begin{array}{c} Q=f_{U}-{\bar{f}}_{U}, \end{array}$$

where  ${\bar{f}}_{U}$ is the remaining force after flexible deformation.

*Q* is the disturbance caused by the flexible deformation of the guide wire. During the operative period, kinetic energy *W* ^1^ is not related to the deflection of the guide wire *v*, so (7) can be simplified as

(9) $$\begin{array}{c} \frac{d}{dt}\left(\frac{\partial W}{\partial \dot{v}}\right)-\frac{\partial W}{\partial v}=Q. \end{array}$$

Equation (4) indicates that *W* is not related to $\dot{v}$:

(10) $$\begin{array}{c} \frac{\partial W}{\partial \dot{v}}=0, \end{array}$$

(11) $$\begin{array}{c} Q=\frac{\partial W}{\partial v}=\frac{1}{2EI}\overset{l}{\underset{0}{\int }}\left(2f_{U}^{2}v\left(x\right)+qf_{U}x^{2}\right)dx. \end{array}$$

It can be shown that

(12) Q=qfUEI[EIfU(l24)sin(l2fUEI)   −(EIfU)3/2sin(l2fUEI)    +EIfUlcos⁡(l2fUEI)+l312],

where $\sin(\left(l/2\right)\sqrt{f_{U}/EI})$ can be regarded as a sine disturbance, and its frequency changes when the length of guide wire *l* changes,

where

(13) $$\begin{array}{c} {\bar{f}}_{U}=f_{U}-Q. \end{array}$$

From (12) and (13), it can be seen that *Q* changes as *l* changes, and it means that the effect of flexible deformation varies as the length of guide wire between the actuator and the operating point varies.

## 3. Control Strategy Design to Suppress the Effect of Flexible Deformation

As there is flexible deformation in the guide wire [7], a control strategy with good robustness is adopted to deal with the model uncertainty. To reduce system lag caused by flexible deformation, the response lag *T*_*d*_ is quantified and SMC control strategy with delayed-output observer is adopted to compensate it.

### 3.1. The *SMC* Control Strategy with Delayed-Output Observer

SMC has the quality of fast response and good transient performance [26, 27]. It can tolerate nonlinear and dynamic uncertainties in a system and guarantee global asymptotic stability [28]. The SMC with delayed-output observer is adopted to deal with the response lag in VR system [23, 29]. For the force feedback control system of virtual surgery in Figure 4, the state equation is built as follows:

(14) x˙(t)=[A+ΔA(t)]x(t)+BfU(t)+f(t)fI(t)=Cx(t),

where Δ*A*(*t*) is the parameter variation and *f*(*t*) is the external disturbance. Consider

(15) $$\begin{array}{c} x\left(t\right)={\left[\begin{array}{cc} f_{I}\left(t\right) & {\dot{f}}_{I}\left(t\right) \end{array}\right]}^{T}. \end{array}$$

The measurement output is

(16) $$\begin{array}{c} f_{I}\left(t\right)=Cx\left(t\right), \end{array}$$

where

(17) C=[10]A=[01−KMMM−BMMM]B=[0ZHMM]T.

It is noted that the *Z*_*H*_ will be simplified by the identification experiment in Section 3.2.

Equation (14) can be rewritten as

(18) x˙(t)=Ax(t)+BfU(t)+d(x,t)fI(t)=Cx(t),

where the generalized disturbance *d*(*x*, *t*) is constructed as

(19) $$\begin{array}{c} d\left(x,t\right)≜\Delta A\left(t\right)x\left(t\right)+f\left(t\right). \end{array}$$

Considering the response lag *T*_*d*_, the output would be

(20) $$\begin{array}{c} {\bar{f}}_{I}\left(t\right)=Cx\left(t-T_{d}\right). \end{array}$$

The error caused by system lags *T*_*d*_ is

(21) f¯I(t)−fI(t)=Cx(t+Td)−Cx(t)=eATd(Cx(t)−Cx(t−Td)).

To compensate the error in (21), the state equation (18) is improved by

(22) x¯˙(t)=Ax¯(t)+BfU(t)+KeATd[fI(t)−C x¯(t−Td)]+d(x,t),

where

(23) $$\begin{array}{c} \bar{x}\left(t\right)={\left[\begin{array}{cc} {\bar{f}}_{I}\left(t\right) & {\dot{\bar{f}}}_{I}\left(t\right) \end{array}\right]}^{T}. \end{array}$$

$\bar{x}(t)$ is the state delayed-output observer, *K* is a constant, and

(24) x¯˙(t−Td)=Ax¯(t−Td)+BfU(t−Td)+K[fI(t)−C x¯(t−Td)]+d(x,t−Td).

Define the observation error as

(25) $$\begin{array}{c} \Delta =\bar{x}\left(t\right)-x\left(t\right). \end{array}$$

From (18) to (25), it can be shown that

(26) $$\begin{array}{c} \dot{\Delta }\left(t-T_{d}\right)=\left(A-KC\right)\Delta \left(t-T_{d}\right). \end{array}$$

The stability requirement of (24) is to select *K* to make the characteristic root of *A* − *KC* in the left half plane. Then, we get

(27) $$\begin{array}{c} \dot{\Delta }\left(t-T_{d}\right)=e^{\left(A-KC)(t-t_{0}\right)}\Delta \left(t_{0}-T_{d}\right), \end{array}$$

where *t*_0_ is the initial time and *e* is the Euler's number. As the characteristic root of *A* − *KC* should be located in the left half plane, so there would be a positive constant *λ*:

(28) $$\begin{array}{c} ||e^{\left(A-KC\right)}||\leq le^{-\lambda }. \end{array}$$

It means that Δ(*t* − *T*_*d*_) is convergent:

(29) $$\begin{array}{c} \underset{t\to \infty }{\lim}\Delta \left(t-T_{d}\right)=0. \end{array}$$

The sliding mode surface is chosen as

(30) $$\begin{array}{c} s=cE+\dot{E}, \end{array}$$

where

(31) c>0E=fE−fI.

And the Lyapunov function is chosen as

(32) $$\begin{array}{c} V=\frac{1}{2}s^{2}. \end{array}$$

The designed control law of sliding mode control is

(33) $$\begin{array}{c} f_{U}\left(t\right)=\frac{1}{k}\left({\ddot{f}}_{E}+a{\dot{\bar{f}}}_{I}+b{\bar{f}}_{I}+\eta \bar{s}+c\dot{\bar{E}}\right), \end{array}$$

where *k*, *a*, and *b* are constants. *f*_*E*_ is the expected force feedback of the system. Consider

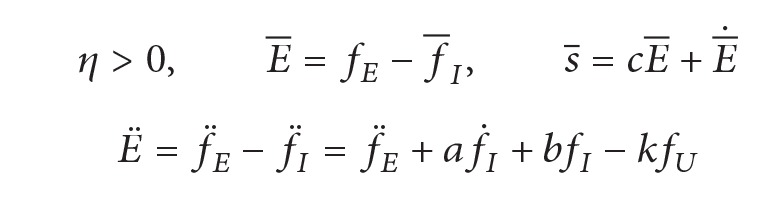
(34)

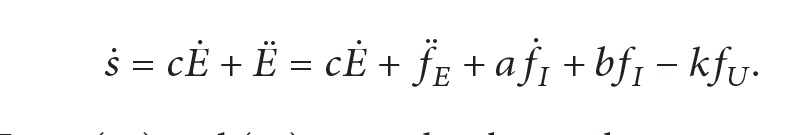
(35)


From (33) and (35), it can be shown that

(36) $$\begin{array}{c} \dot{s}=-\eta s+\left(b-\eta c\right){\overset{⌢}{f}}_{I}+\left(a-\eta -c\right){\overset{⌢}{\dot{f}}}_{I}, \end{array}$$

where

(37) $$\begin{array}{c} {\overset{⌢}{f}}_{I}=f_{I}-{\bar{f}}_{I}\\ \dot{V}=\dot{s}s=-\eta s+s\left[\left(b-\eta c\right){\overset{⌢}{f}}_{I}+\left(a-\eta -c\right){\overset{⌢}{\dot{f}}}_{I}\right]. \end{array}$$

Because the observer is convergent,

(38) $$\begin{array}{c} \underset{t\to \infty }{\lim}{\overset{⌢}{f}}_{I}=0,\;\;\underset{t\to \infty }{\lim}{\overset{⌢}{\dot{f}}}_{I}=0. \end{array}$$

It can be shown that

(39) $$\begin{array}{c} \dot{V}\leq 0. \end{array}$$

It means that the control law satisfies Lyapunov stability.

### 3.2. System Identification

**Figure 6:**
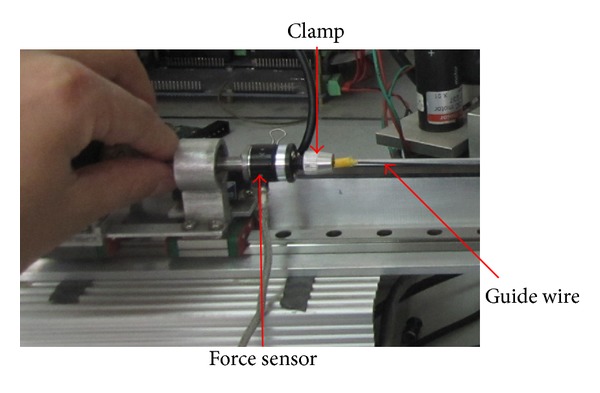
The force feedback measurement device.

Equation (3) describes the relationship between the actuator force *f*_*U*_ and the measured force *f*_*I*_. The measurement device of the force feedback at the operating point is shown in Figure 6.

A sensor measures the force feedback in real-time. The sensor is hollow and the guide wire passes through it. The proposed device is suitable to measure a large range of motion of deformable tools, such as the guide wire.

To model the parameter variation of the control system, the process model in system identification tool of matlab is adopted to simulate the force transmission of the guide wire. Consider

(40) $$\begin{array}{c} f_{I}=P_{n}f_{U}+\Delta e, \end{array}$$

where *P*_*n*_ is the plant model, Δ is the *ARMA* disturbance model, and *e* is white noise.

**Figure 7:**
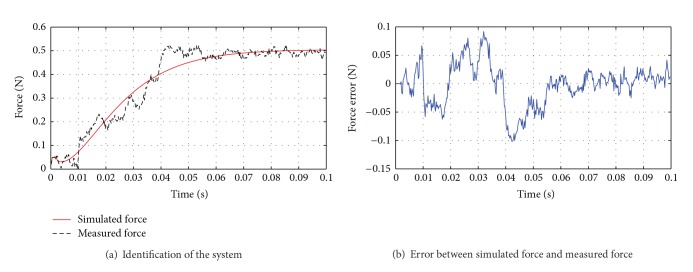
Identification of the force feedback control system.

The response process of a constant force after transmission via guide wire is measured, as shown in Figure 7(a). The black dotted line is the force measured by the sensor, and red solid line is the force of simulated model. Parameters of the system model in (40) are as follows:

(41) $$\begin{array}{c} P_{n}=\frac{1.0101}{0.000155027401s^{2}+0.024902s+1}e^{-0.0024s}, \end{array}$$

(42) $$\begin{array}{c} \Delta =\frac{s+2380}{s+19.25}. \end{array}$$

From (41), it is shown that

(43) $$\begin{array}{c} \frac{M_{M}}{K_{M}}=0.000155,\;\;\frac{B_{M}}{K_{M}}=0.0249,\\ \frac{Z_{H}}{K_{M}}=1.0101. \end{array}$$

Then *A* and *B* in (14) are as follows:

(44) A=[01−160−6450],B=[06515]T.

In the identification, the loss function is 8.01527 × 10^−5^ N and the Akaike final prediction error is 8.21565 × 10^−5^ N. Estimated by the error between simulated force and measured force in Figure 7(b), the maximum amplitude of perturbation was 0.11 N.

### 3.3. The Performance of the *SMC* Strategy in the Control System

In VR training, a virtual force model is established to give feedback force [30–32]. In other words, it should give the user a sense of fidelity by stimulus-response relation [33–35]. Studies showed that an operator can use the tools expertly depending on appropriate feeling of mass and stiffness [36–39].

Research reveals that the real time force feedback needs a refresh rate of more than 300 HZ in order to achieve realistic requirements [40]. In this study, it is required that the response lag cannot exceed 3.3 ms (the period when refresh rate is 300 HZ).

**Figure 8:**
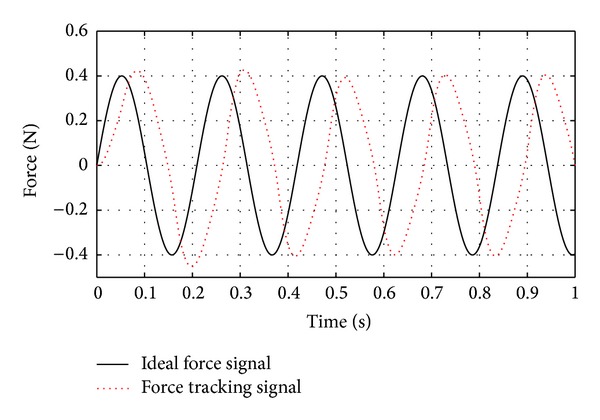
Performance of force feedback control when using regular SMC. The disturbance is sine wave plus random interference (the amplitude is 0.11 N).

Control performance of regular SMC strategy is shown in Figure 8.

**Figure 9:**
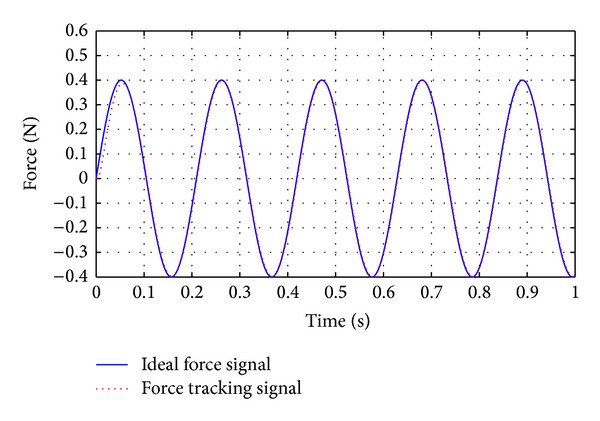
Performance of force feedback control when using SMC with delayed-output observer. The disturbance is sine wave plus random interference (the amplitude is 0.11 N).

Under the control of regular SMC strategy, the response lag is about 48 ms. The SMC with delayed-output observer is used to suppress the response lag, seen in Figure 9. In (22), the response lag is determined as *T*_*d*_ = 48 ms. To be better convergent, the parameter *K* is designed as

(45) $$\begin{array}{c} K={\left[\begin{array}{cc} 0.0001 & 0.0001 \end{array}\right]}^{T}. \end{array}$$

Comparing Figure 9 with Figure 8, it can be seen that SMC with delayed-output observer can eliminate the response lag caused by flexible deformation of the guide wire. The response lag is below 3.3 ms when the SMC with delayed-output observer is adopted, as shown in Figure 9.

As it is discussed in Section 2.2, the disturbance caused by the flexible deformation of the guide wire is related to the length of the guide wire, the performance of the force feedback control is assessed in Section 4 when the length of the guide wire changes.

## 4. Performance Assessment of the Force Feedback Control

The controller design focuses on development of the control strategy and its application, while the performance assessment is concerned about whether the designed controller is in accordance with the required performance at the operating stage [41]. The assessment of the current controller generally includes determination of the capability of the control system, design of a benchmark for performance assessment, assessment of the performing loops, diagnosis of the underlying causes, and improvements suggestion [42].

Various assessment methods are used in surgical training [43]. The reliability and validity of the methods should be examined. Reference [44] has assessed the hemodynamic of virtual surgery, it reveals the importance of surgical planning and multiparameter patient-specific modeling in complex congenital heart disease. This study focuses on the force feedback control system in virtual surgery of minimally invasive vascular intervention.

To assess performance of virtual vascular intervention surgery, a specific control performance metric for the virtual surgery is needed. Reference [45] presented robust methods for performance of virtual diagnostic hysteroscopy, and a clinical study is carried out to investigate the implemented performance metrics. In our force feedback control system, the requirement of fidelity of the virtual vascular intervention surgery is to reduce the control force error between the virtual and real surgery. The root mean square error is used to evaluate the simulation of the force feedback device [46]. In this study, variance is used to assess the performance of the force feedback control. And the theoretic minimum variance is chosen as a benchmark for the performance assessment [47].

### 4.1. The Minimum Variance Performance Benchmark

**Figure 10:**
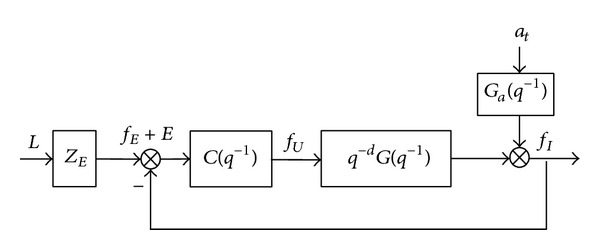
The force feedback control system.

In this section, a minimum variance controller is designed and the associated minimum variance performance will be found for the control system of virtual vascular intervention surgery [48]. The force feedback control system is described in Figure 10.

As shown in Figure 10, *q* ^−*d*^ is a *d*-step time delay of the controller. In the VR simulator, *d* = 10. *a*_*t*_ is white noise with zero mean and its variance is *σ*_*a*_^2^ = 8∗10^−5^.

In Section 2.2, it can be seen that the output of the control system *f*_*I*_ is related to *f*_*U*_ and *f*_*U*_^1/2^. The control system can be described as

(46) $$\begin{array}{c} f_{I}=G_{1}f_{U}+G_{2}f_{U}^{1/2}+G_{a}a_{t}. \end{array}$$

The control input is defined as

(47) $$\begin{array}{c} f_{U}=-C_{1}f_{I}-C_{2}f_{U}^{1/2}. \end{array}$$

So the minimum variance of output can be obtained by the coefficients *C*_1_ and *C*_2_:

(48) $$\begin{array}{c} {\mathrm{Var}\{f_{I}\}|}_{mv}∶=\underset{C_{1},C_{2}}{\min}\mathrm{Var}\left\{f_{I}\right\}. \end{array}$$

From (46) and (47), it can be seen that

(49) $$\begin{array}{c} f_{I}={\left(I+G_{1}C_{1}\right)}^{-1}\left[\left(G_{2}-G_{1}C_{2}\right)f_{U}^{1/2}+G_{a}a_{t}\right]. \end{array}$$

*G*_1_ and *G*_*a*_ are decomposed as follows:

(50) G1=G¯1+q−dG¯¯1G2=G¯2+q−dG¯¯2Ga=G¯a+q−dG¯¯a.

Equation (41) indicates that the system is a pure delay process, so

(51) G¯1=0G¯2=0.

Then, (49) is equivalent to

(52) fI=(I+q−dG¯¯1C1)−1×((G2−G1C2)fU1/2+G¯aat+q−dG¯¯aat),

as it can be derived that

(53) $$\begin{array}{c} \left(I+q^{-d}{\bar{\bar{G}}}_{1}C_{1}\right)\left(I-q^{-d}{\left(I+q^{-d}{\bar{\bar{G}}}_{1}C_{1}\right)}^{-1}{\bar{\bar{G}}}_{1}C_{1}\right)=I. \end{array}$$

From (52) and (53),

(54) fI=(I−q−d(I+q−dG¯¯1C1)−1G¯¯1C1) ×(q−d(G¯¯2−G¯¯1C2)fU1/2+G¯aat+q−dG¯¯aat)=G¯aat+q−d(I+q−dG¯¯1C1)−1 ×[(G¯¯a−G¯¯1C1G¯a)at+(G¯¯2−G¯¯1C2)fU1/2].

If we design

(55) C1=G¯¯1 −1G¯¯aG¯a −1C2=G¯¯1 −1G¯¯2,

it can be shown from the output with minimum variance from (46) to (53) that

(56) $$\begin{array}{c} {f_{I}|}_{mv}={\bar{G}}_{a}a_{t}. \end{array}$$

From (42), ${\bar{G}}_{a}$ can be shown by the *ARMA* model as

(57) $$\begin{array}{c} {\bar{G}}_{a}=\frac{1+1.357q^{-1}}{1-0.9809q^{-1}}. \end{array}$$

The *d*-step ahead predictive model of the force feedback control system is as follows:

(58) fI(t+d)=G¯¯1fU(t)+G¯¯2fU1/2(t)+G¯aat(t+d)+G¯¯aat(t).

The mathematical expectation of *y*_*t*+*d*_ has following form [47, 49]:

(59) $$\begin{array}{c} {\bar{\bar{f}}}_{I}\left(t+d\right)={\bar{\bar{G}}}_{1}f_{U}+{\bar{\bar{G}}}_{a}a_{t}\left(t\right). \end{array}$$

Then, the model prediction error can be shown as

(60) $$\begin{array}{c} E\left(t+d\right)=f_{I}\left(t+d\right)-{\bar{\bar{f}}}_{I}\left(t+d\right)={\bar{G}}_{a}a_{t+d}. \end{array}$$

When using the minimum variance controller,

(61) $$\begin{array}{c} {\bar{\bar{f}}}_{I}\left(t+d\right)=0. \end{array}$$

The output *f*_*I*|*mv*(*t*+*d*)_ is only dependent on the *d*-step forward calculations [50, 51]. Consider

(62) fI|mv(t+d)=G¯aat(t+d)=(1+Ga,1q−1+⋯Ga,d−1qd+1)at(t+d).

It is equivalent to

(63) fI|mv=G¯aat(t+d)=(1+Ga,1q−1+⋯Ga,d−1qd+1)at(t).

The minimal variance of output is as follows:

(64) $$\begin{array}{c} {\mathrm{Var}\left\{f_{I}\right\}|}_{mv}=\overset{d-1}{\underset{j=0}{\sum }}G_{a,j}\overset{2}{\underset{a}{\sum }}G_{a,j}^{T}. \end{array}$$

We can see that the output variance Var⁡{*f*_*I*_} reaches the minimal value, which is independent of the controller. But in practical engineering, the controller is usually not minimal variance controller. The control performance index is defined as follows [41, 48]:

(65) $$\begin{array}{c} \eta =\frac{{\mathrm{Var}\left\{f_{I}\right\}|}_{mv}}{\mathrm{Var}\left\{f_{I}\right\}}. \end{array}$$

The advantage of this performance index is that minimal variance benchmark can be calculated from routine operating data by estimating the impulse response from noise-to-output transfer function. This definition of the controller performance index satisfies 0 ≤ *η* ≤ 1. The distance between minimum variance control system and actual control system can be directly seen by *η*. The value *η* = 1 indicates the ideal case of minimum variance control, whereas *η* = 0 shows the case of the worst control.

### 4.2. The Performance of the *SMC* Control Strategy with Delayed-Output Observer in the Force Feedback Control

**Figure 11:**
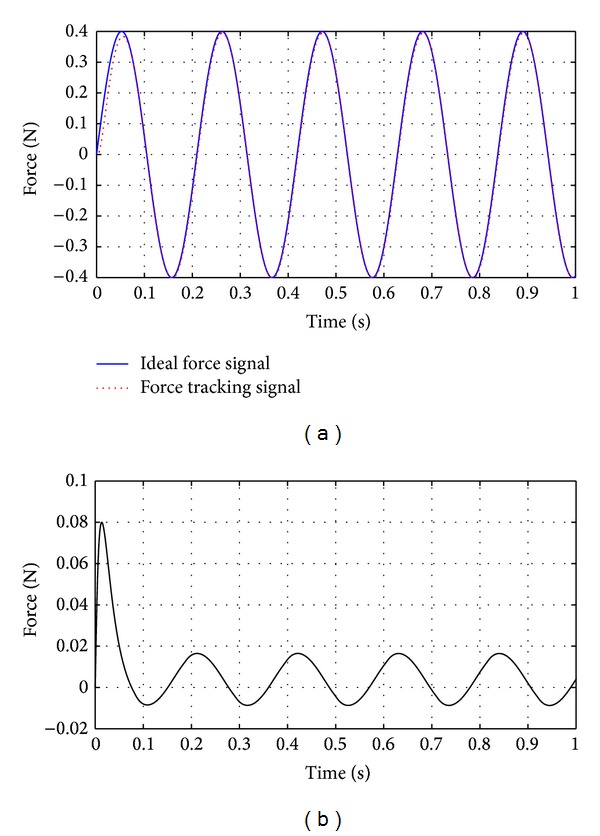
Force feedback control when length of the guide wire between actuator and the operating point is 8 cm.

**Figure 12:**
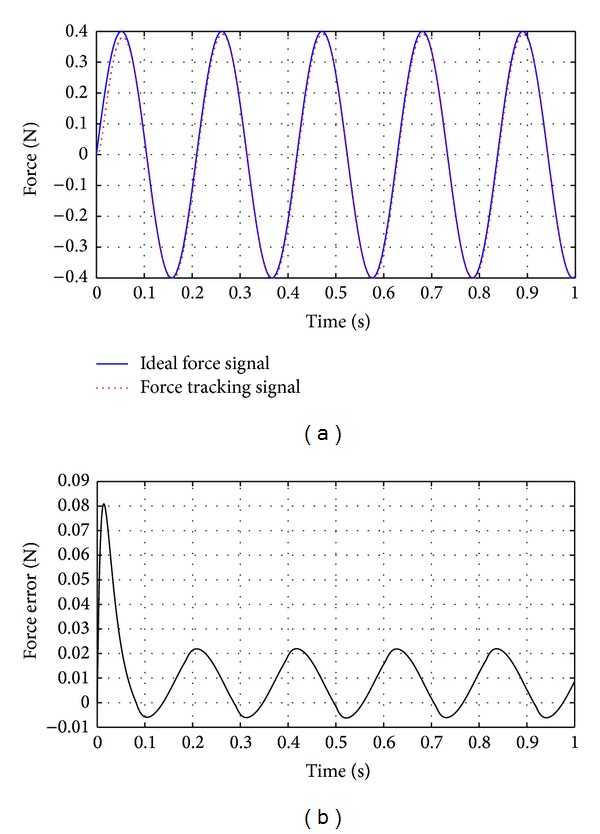
Force feedback control when length of the guide wire between actuator and the operating point is 18 cm.

As shown in (11) in Section 2.2, the disturbance caused by flexible deformation is related to the the length of guide wire between the actuator and operating point of human hand. So we compare the control performance under two different conditions: one condition is when length of guide wire is 8 cm, while the other is when length of guide wire is 18 cm, as shown in Figures 11 and 12.

As shown in Figure 11, the maximum control error is below 0.02 N when the length of guide wire is 8 cm, while Figure 12 indicates that the maximum control error exceeds 0.02 N when the length of guide wire is 18 cm. It reveals that the control error will change with length of the guide wire. The force feedback control has big error in the initial stage, which is approximately 0.08 N. The above error is because the system lag has not been compensated efficiently in the initial period. As the time exceeds 0.1 s, the control error is stable because system lag has been suppressed.

**Figure 13:**
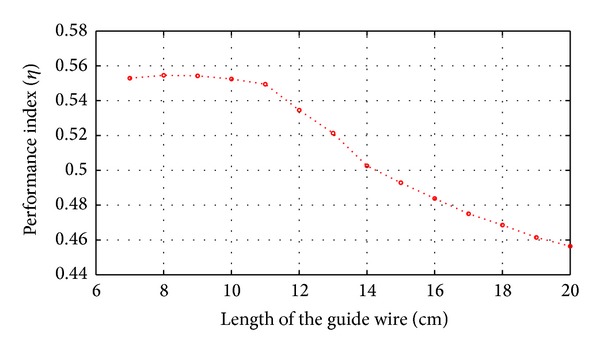
Performance index of the force feedback control system when the length of guide wire changes.

As shown in Figure 13, when 7 cm ≤ *L* ≤ 11 cm, the performance index of the force feedback control ranges from 0.54 to 0.56, which is acceptable. When *L* ≥ 12 cm, the performance index reduces steeply as *L* is getting larger, which means that the performance of the force feedback control decreases.

### 4.3. Discussion

According to the specification for structural design of virtual surgery, the length of guide wire *L* has the lower boundary of 8 cm. Figure 13 indicates that the optimal length of the guide wire between actuator and the operating point ranges from 8 cm to 11 cm. Performance assessment approach proposed in this paper has great meaning in the design process of force feedback device. The performance index and optimal displacement of Actuator 1 and Actuator 2 and Sctuator 2 and Sctuator 3 in Figure 2 can be shown in the same way.

Based on the performance index, it can be seen that the control system can be further improved. As we can see from Figures 11 and 12, the force control error is bigger in the initial period of the force feedback control. The control strategy needs to be improved to reduce the force control error at the initial period. As the operating speed is different among users, the transition rate of the feedback force varies. It would generate different control accuracy. In the future, we will design the control strategy to improve the control performance when the simulator is operating at the usual speed.

## 5. Conclusion

In virtual surgery of minimally invasive vascular intervention, the response lag caused by flexible deformation of the guide wire would affect the fidelity of the force feedback control system. In this study, the SMC with delayed-output observer is adopted to eliminate the effects of flexible deformation. The whole control strategy is easy to implement and is well accepted in the field.

In our force feedback control system, the requirement of fidelity of the virtual vascular intervention surgery is to reduce the control force error between the virtual and real surgery. The minimum variance is chosen as the performance benchmark to assess the control performance of the virtual surgery. The results of performance assessment reveals the optimal length of guide wire between actuator and operating point. And this method can be used to assess other lengths of surgical instruments in the VR simulator.

As shown in Figures 11 and 12, the SMC with delayed-output observer has the control error in initial period. In future research, the control strategy will be improved to reduce the initial control error.
